# Discordance of vancomycin minimum inhibitory concentration for methicillin-resistant Staphylococcus aureus at 2 *μ*g/mL between Vitek II, *E*-test, and Broth Microdilution

**DOI:** 10.7717/peerj.8963

**Published:** 2020-05-11

**Authors:** Chien-Feng Kuo, Chon Fu Lio, Hsiang-Ting Chen, Yu-Ting Tina Wang, Kevin Sheng-Kai Ma, Yi Ting Chou, Fu-Chieh Chang, Shin-Yi Tsai

**Affiliations:** 1 Division of Infectious Disease, Mackay Memorial Hospital, Taipei, Taiwan; 2 Department of Laboratory Medicine, Mackay Memorial Hospital, Taipei, Taiwan; 3 Department of Medicine, Mackay Medical College, Taipei, Taiwan; 4 Department of Pathology, School of Dental Medicine, University of Pennsylvania, Philadelphia, USA; 5 Infection control center, Mackay Memorial Hospital, Taipei, Taiwan; 6 Department of Health Policy and Management, Bloomberg School of Public Health, Johns Hopkins University, Baltimore, Maryland, U.S.; 7 Graduate Institute of Long-Term Care, Graduate Institute of Biomedical Sciences, Mackay Medical College, New Taipei City, Taiwan

**Keywords:** Methicillin-resistant Staphylococcus aureus (MRSA), Clinical and Laboratory Standards Institute (CLSI), Microbial automatic identification system (Vitek II), *E*-test, Broth Microdilution, Minimal inhibitory concentration (MIC ), The inter-test agreements, Hospital-acquired MRSA, Community-acquired MRSA, Antimicrobial stewardship

## Abstract

**Background:**

Vancomycin, the first line antibiotic for methicillin-resistant *Staphylococcus aureus* (MRSA) bacteremia, is often administered inappropriately when MIC is greater than 2 µg/mL, including ‘susceptible’ strains. This study assessed the discordance of vancomycin minimum inhibitory concentration (MIC) for methicillin-resistant* Staphylococcus aureus* (MRSA).

**Methods:**

In total, 229 MRSA isolates from blood cultures collected between 2009 and 2015 at a tertiary hospital in Taiwan were examined. The MICs of vancomycin were measured using Vitek 2, *E*-test, and standard broth microdilution at the level of 2 µg/mL.

**Results:**

The geometric mean of the MICs of hospital-acquired MRSA was higher than that of community-acquired MRSA (*P* < 0.001), with the exact agreement rates (with broth microdilution) at 2 µg/mL being 53.6% in Vitek 2 and 86.7% in *E*-test. Overall, *E*-test (98.1%) had more categorical accordance than did Vitek 2 (94.0%; *P* = 0.026). Vitek 2 had a tendency to overestimate MRSA in high-MIC isolates, whereas E-test inclined underestimation in low-MIC isolates. Surprisingly, the discordance rates of MRSA vancomycin MICs were higher in hospital-acquired isolates (13.3%–17.0%) than in community-acquired isolates (6.2%–7.0%).

**Conclusion:**

The Infectious Diseases Society of America recommends the use of alternative antimicrobial agents when vancomycin MIC is ≥ 2 µg/mL; in this study, only 53.6% of the isolates tested using Vitek 2 showed a high MIC in the broth microdilution method. Accurate identification of the resistance profile is a key component of antimicrobial stewardship programs. Therefore, to reduce inappropriate antibiotic use and mitigate the emergence of resistant strains, we recommend using complementary tests such as *E*-test or Broth microdilution to verify the MIC before administering second-line antibiotics.

**Strengths:**

(1) We compared the categorical agreement between different methods measuring MRSA MICs level. (2) Physicians should incorporate this information and consider a complementary test to verify the appropriateness of the decision of shifting vancomycin to second-line antibiotic treatment to improve patients’ prognosis. (3) MRSA-vancomycin MICs at a cutoff of 2 µg/mL obtained using Vitek II exhibited a higher sensitivity level and negative predictive value than those obtained using *E*-test in the prediction of categorical agreement with standard broth microdilution.

**Limitation:**

(1) Our research was based on a single hospital-based study. (2) The MRSA strains in this study were stored for more than 12 months after isolation. (3) We did not collect information on clinical prognosis.

## Introduction

*Staphylococcus aureus* is a common bacterium and opportunistic pathogen that thrives on skin and mucosal surfaces, and often can cause a range of mild to severe infections if it enters the body through breaks in these surfaces. Unfortunately, this pathogen has been building up resistance to conventional penicillins because of the widely inappropriate use of penicillin. Although an increase in methicillin-resistant *S. aureus* (MRSA) vancomycin minimum inhibitory concentration (MIC) has been observed (Robert, Bismuth & Jarlier, 2006; Wang et al., 2006; Steinkraus, White & Friedrich, 2007), vancomycin remains the most commonly prescribed antimicrobial agent to inhibit MRSA infection in many countries (Levine, 2006).

Empirical studies have shown that there have been a higher mortality associated with MRSA bacteremia when vancomycin was used for treatment of infections with high vancomycin MIC strains (Sakoulas et al., 2004; Soriano et al., 2008; Yoon et al., 2010; Aguado et al., 2011; Holmes et al., 2013; Jacob & DiazGranados, 2013; Chen et al., 2014). A meta-analysis suggested that the mortality related to MRSA infection is correlated with high vancomycin MICs (>1.5 µg/mL) (Van Hal, Lodise & Paterson, 2012). This finding may be attributed to failure to reach optimal pharmacokinetic targets (Holmes et al., 2013); however, this idea remains controversial (Castaneda et al., 2017). Recent evidence suggests that vancomycin is not the most appropriate antibiotic to be administered for MRSA-vancomycin MICs ≥2 µg/mL, and alternative anti-MRSA agents have been suggested (Lewis 2nd & Ellis, 2007; Liu et al., 2011). From a technical standpoint, automated machines yield a “susceptible” result in a range of three MRSA-vancomycin MICs, specifically 0.5, 1.0, and 2.0 µg/mL. Clinical results may not be favorable as physicians prescribe vancomycin solely based on these automated susceptibility tests which could fail to provide accurate MIC at those specific breakpoints.

Broth microdilution or agar dilution had previously been the gold standard for determining MIC. However, both methods became time-consuming and were rendered somewhat obsolete due to the rise of automated machines such as Vitek II. Therefore, the aim of this study was to investigate the agreement of MIC results between VITEK^®^ 2 AST, *E*-test, and broth microdilution, in order to clarify whether a complementary broth microdilution test would be necessary to confirm the accuracy of MRSA-vancomycin MICs at a cutoff of 2 µg/mL, and whether clinicians should consider other antibiotics regimen.

## Methods

### Study design and data collection

This retrospective study was conducted at and approved by the Institutional Review Board of Mackay Memorial Hospital, a tertiary referral center in Taipei, Taiwan (18MMHIS063). Patient data, namely MRSA bacteremia from 2009 to 2015, was retrieved from the microbiology laboratory databases of the hospital. This study focused on a cross comparison of the MICs of invasive MRSA initially detected with Vitek II. Therefore, we examined the inter-test agreements between the initial Vitek II automated identification method, *E*-test, and conventional broth microdilution at various MIC cutoffs.

### MIC determination

All strains were isolated according to routine procedures. After initial culture, the strains were stored in an ultra-low-temperature refrigerator at −80 °C. All samples were sub-cultured twice before MIC testing. The MRSA strain was first inoculated on Luria–Bertani broth, then cultured in an incubator for 24 h, and finally transferred to a colistin plus nalidixic acid medium. Vitek II (bioMérieux, Hazelwood, MO) and *E*-test MICs were determined using a standard inoculum (turbidity equivalent to 0.53–0.65 McFarland standard). Broth microdilution was conducted according to the CLSI guidelines, 30th Edition (CLSI, 2020), and vancomycin concentrations were measured as 0.25, 0.5, 1.0, 2.0, 4.0, and 8.0 µg/mL. Susceptibility is defined by a MIC of ≤2 µg/mL. Isolates with a MIC range of 4–8 µg/mL were considered intermediate, and those with a MIC of ≥16 µg/mL were considered resistant(2017). All MRSA-vancomycin MICs were determined using different testing methods by experienced laboratory technicians by following the standard operating procedures provided by manufacturers. The AST-P622 drug sensitivity card was used to detect drug sensitivity of Staphylococcus species. When *Staphylococcus species* were tested twice to be resistant to Vancomycin, Vancomycin *E*-test was also performed using *S. aureus* ATCC29213 QC strain. It is only when *E*-test showed the same result that the report of “intermediate” or “resistance” to Vancomycin by VITEK 2 and AES was released. We divided the MRSA strains into two groups: community-acquired infection and hospital-acquired infection. According to the criteria provided by the American Centers for Disease Control and Prevention in 2000, community-acquired MRSA is defined as infection confirmed within 48 h of hospitalization, and hospital-acquired MRSA is defined as infection detected by a positive culture taken more than 48 h after hospital admission and treated in the inpatient setting (Prevention,, NCEZID).

### Statistical analysis

We compared the geometric me complementary tests values among different testing methods via one-way analysis of variance(ANOVA). We examined the categorical agreement between the three testing methods as isolates that had concordant results to determine high (≥2 µg/mL) and low (<2 µg/mL) MRSA-vancomycin MICs (Chen et al., 2014). The chi-square test was adopted to determine the significant difference between two groups. Pearson’s correlation analysis was conducted to describe the linear dependence between the two testing methods. R coefficients are also presented. In all the comparisons, a *P* value of <0 .05 was considered statistically significant. Statistical analyses were performed with SPSS software, Version 17.0 (SPSS Inc., Chicago, IL, USA).

## Results

A total of 229 strains of MRSA were collected for this study, in which 136 were the community infection type and 93 were the nosocomial infection type. Our results obtained from the micro-dilution test indicated that among the 136 community-type MRSA, 80 strains showed a vancomycin MIC of 0.5 µg/mL, 48 strains showed a MIC of 1.0 µg/mL, and 8 strains showed a MIC of 2.0 µg/mL. Among the 93 nosocomial-type MRSA strains, 25 showed a vancomycin MIC of 0.5 µg/mL, 48 showed a MIC of 1.0 µg/mL, and 20 showed a MIC of 2.0 µg/mL (Table 1).

**Table 1:** Vancomycin MIC of MRSA isolates, include 136 community-type and 93 nosocomial-type, were detected by *Microdilution*.

**Methods**	**No.**	MIC = 0.5 **(*μ* g/mL)**	**MIC = 1** **(*μ* g/mL)**	**MIC ≥ 2** **(*μ* g/mL)**		
-Community-type -Nosocomial-type Total	136 93 229	80 25	48 48	8 20		

**Notes.**

The total 229 MRSA-vancomycin MIC were measured by microdilution as standard method and subgroup into community and nosocomial-type. After subculture process, 13 strains of MIC ≥2 µg/mL were excluded due to poor quality control.

**Figure 1:**
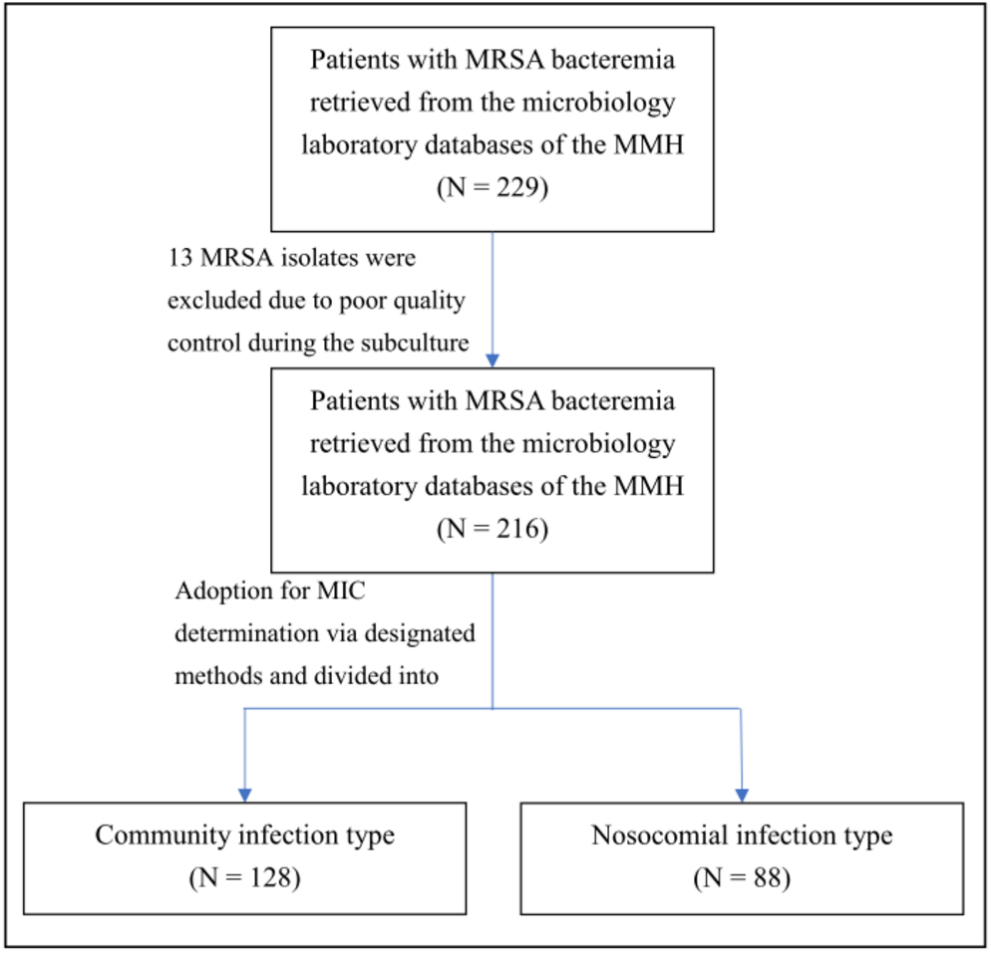
Flow diagram of participants. A total of 229 primary care MRSA isolates from blood cultures collected between 2009 and 2015 and 216 isolates further completed the study. Patients with MRSA bacteremia retrieved from the microbiology laboratory databases, and 13 MRSA isolates were excluded due to poor quality control during the subculture.

After 13 MRSA isolates were excluded due to poor quality control during the subculture process, 216 strains of *S. aureus* were qualified and thus were adopted for MIC determination via designated methods (Fig. 1). Among these methods, Vitek II identification yielded the highest geometric mean of MRSA-vancomycin MICs (0.91 ±  0.48) and the greatest proportion of MRSA isolates, with a vancomycin MIC as high as ≥2 µg/mL (28/216, 13.0%; Table 2). However, no statistical difference in geometric mean could be ascertained between groups (*P* > 0.05).

**Table 2:** Vancomycin MIC of MRSA isolates detected by VITEK-II, *E*-test, and Microdilution.

**Methods**	**No.**	**MIC = 0.5** (*μ* g/mL)	**MIC = 1** **(*μ* g/mL)**	**MIC ≥ 2** (*μ* g/mL)	**Geometric** mean (*μ***g/mL)**	*P* value^a^
VITEK-II	216	93	95	28	0.91 ± 0.48	0.116
*E*-test	216	100	101	15	0.84 ± 0.40	
Microdilution	216	98	103	15	0.84 ± 0.40	

**Notes.**

a Comparison of geometric mean value of MICs between different testing methods by one-way analysis of variation.

**Figure 2:**
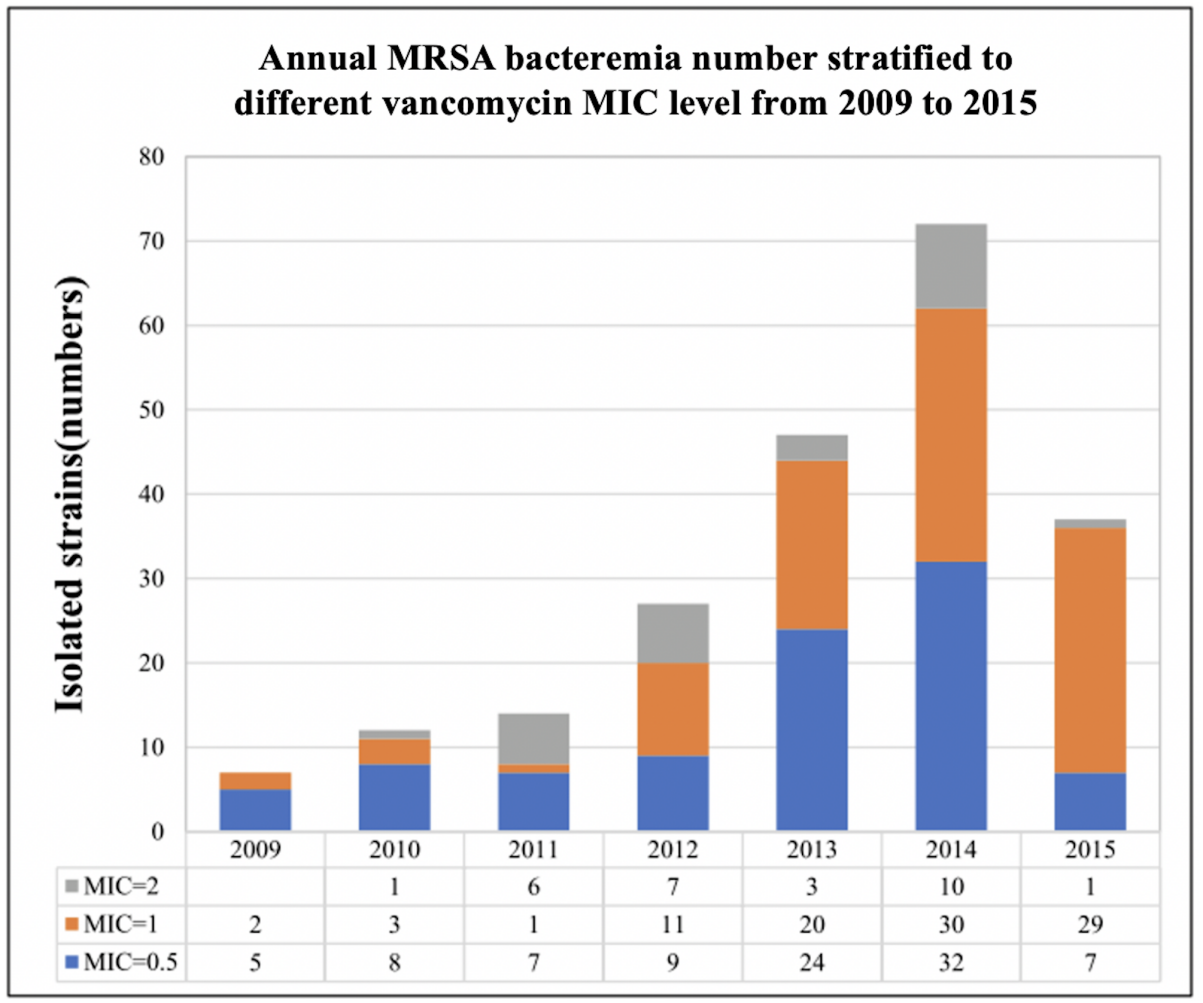
Statistic of annual case no. of invasive MRSA from 2009 to 2015. There was an upward trend of invasive MRSA bacteremia case from 2009 to 2014. This surge was ceased since 2015 but the percentage of MRSA with MIC ≥ 1 *μ*g/mL was still high (81.1%). The above MICs were measured by VITEK-II.

Additionally, an increasing prevalence of invasive MRSA from 2009 to 2014 was suggested by our findings (Fig. 2). This surge has apparently plateaued since 2015, but the percentage of MRSA with a MIC of ≥1 µg/mL has remained high (81.1%).

A total of 216 *S. aureus* strains were further divided into three groups according to the following vancomycin MICs: 2.0, 1.0, and 0.5 µg/mL. This study explored the agreement between the three different methods (Vitek II automated identification, *E*-test, and broth microdilution), which was defined as a MIC variation within ±1 log2 with broth microdilution as the reference standard (Chen et al., 2014) as presented in Table 3. Regarding the exact agreement at the critical value with a MIC of 2 µg/mL, both the Vitek II (53.6%) and *E*-test (86.7%) methods were not reflective of the actual extent. Notably, greater discordance in MRSA-vancomycin MICs was observed in hospital-acquired isolates (13.3%–17%) than in community-acquired isolates (6.2%–7.0%). Nonetheless, the essential agreement between Vitek II and broth microdilution, as well as that between *E*-test and broth microdilution, were 100% across all MRSA-vancomycin MIC levels.

**Table 3:** Determining the differences and agreements of MRSA-vancomycin MICs between automated susceptibility testing, *E* test and standard microdilution method in 216 MRSA bloodstream isolates.

	−1	Same^a^	+1	+2	**Exact agreement** (*N*, %)	**Essential agreement**^b^ (N, %)
All ioslates (*N* = 216)						
***Broth microdilution***		Ref			Ref	Ref
*VITEK-II*						
MIC = 0.5 (µg/mL)	3	90	0	0	90 (96.8%)	93 (100%)
MIC = 1.0 (µg/mL)	0	87	8	0	87 (91.6%)	95(100%)
MIC = 2.0 (µg/mL)	0	15	13	0	15 (53.6%)	28 (100%)
***Etest***						
MIC = 0.5 (µg/mL)	9	91	0	0	91 (91.0%)	100 (100%)
MIC = 1.0 (µg/mL)	2	92	7	0	92 (91.1%)	101 (100%)
MIC = 2.0 (µg/mL)	0	13	2	0	13 (86.7%)	15 (100%)
**Community-acquired isolates (*N* = 128)**						
***VITEK-II***	1	119	8	0	119 (93.0%)	128 (100%)
***Etest***	4	120	4	0	120 (93.8%)	128 (100%)
**Hospital-acquired isolates (*N* = 88)**						
***VITEK-II***	2	73	13	0	73 (83.0%)	88 (100%)
***Etest***	7	76	5	0	76 (86.4%)	88 (100%)

**Notes.**

a Number of MRSA isolates for which the MICs determined by automated methods differed from the Broth microdilution MICs by the scale of log2 dilutions.

b The agreement of MICs’ variation within ±1 log2 between automated methods, *E* test and the standard Broth microdilution method.

**Figure 3:**
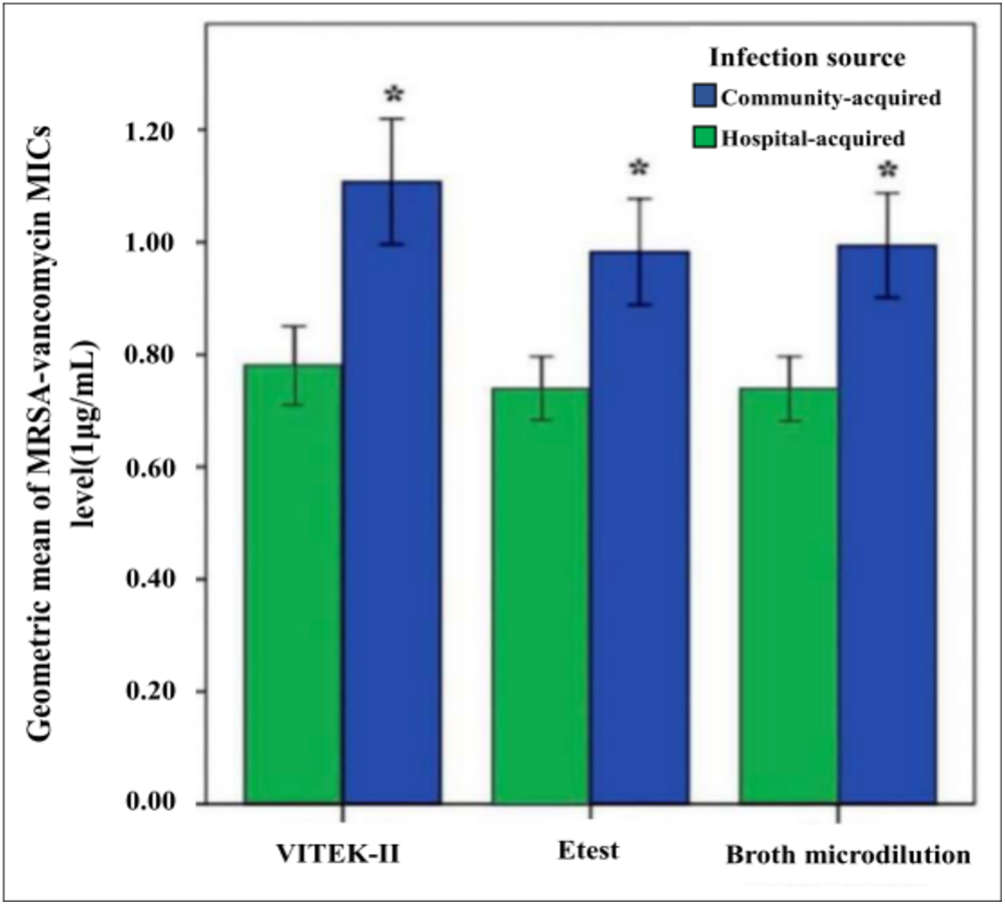
Comparison of geometric mean of MRSA-vancomycin MICs between community and hospital-acquired isolates by different methods. Results suggested there are significant higher mean MICs in hospital-acquired MRSA compared with community-acquired, regardless of the type of detecting systems (All *P* < .001). The above MICs were measured by VITEK-II, *E*test, and Broth microdilution responsively.

Since MIC = 2 µg/mL is an indicator for shifting vancomycin to second-line antibiotic treatment, investigating the categorical accordance would provide clinical significance. In this sense, *E*-test (98.1%) yielded a more accurate and reliable result compared to Vitek II (94.0%); moreover, this difference in terms of the accuracy was statistically significant (*P* = 0.026; Table 4). The main reason for this may be that after the infection source was analyzed, Vitek II was determined to yield a relatively inaccurate MIC determination in hospital-acquired isolates (89.8%) compared with *E*-test (97.7%). No evident difference in categorical agreement was observed between the Vitek IIs(96.9%) and the *E*-tests (98.4%) in community-acquired isolates (*P* = 0.409). Because higher MICs were associated with lower accuracy levels in both methods, the most logical explanation for this was that the MIC values in hospital-acquired MRSA were typically higher (Fig. 3). Specifically, hospital-acquired MRSA exhibited significantly higher mean values of MICs compared to community-acquired MRSA, regardless of the adopted detection systems (All *P* < 0 .001). All in all, *E*-tests exhibited a stronger linear relationship with broth-microdilution-detected-MICs (Pearson’s coefficient: 0.868–0.883) than Vitek IIs (Pearson’s coefficient: 0.785–0.865) in Table 4.

**Table 4:** Categorical agreement and Pearson correlation of MRSA-vancomycin MICs between automated testing methods and standard Microdilution method.

	**Categorical agreement**^a^ **(N, %, *P* value)**			**Pearson’s correlation** **(R coefficient, *P* value)**	
	Broth microdilution	VITEK-II	*P* value^b^	Broth microdilution	VITEK-II
**All ioslates (*N* = 216)**					
VITEK-II	203 (94.0%)	–	0.026	0.840 (<0.001)	–
Etest	212 (98.1%)	203 (94.0%)		0.883 (<0.001)	0.775 (<0.001)
**Community-acquired isolates (*N* = 128)**					
VITEK-II	124 (96.9%)	–	0.409	0.865 (<0.001)	–
Etest	126 (98.4%)	122 (95.3%)		0.875 (<0.001)	0.835 (<0.001)
**Hospital-acquired isolates ( *N* = 88)**					
VITEK-II	79 (89.8%)	–	0.029	0.785 (<0.001)	–
Etest	86 (97.7%)	81 (92.0%)		0.868 (<0.001)	0.681 (<0.001)

**Notes.**

a Categorical agreement refers to having concordant results when determining high (≥2 µg/mL) and low (<2 µg/mL) vancomycin MICs.

b Chi-Square test was used to determine the difference in categorical agreement between VITEK-II and *E* test groups.

## Discussion

This study emphasized the agreement of MIC results among various methods. One of our most significant findings was that at a cutoff of 2 µg/mL, only 53.57% of positive vancomycin MICs measured by Vitek II were consistent with the actual high MICs determined by broth microdilution. This implies that nearly 47% of patients might receive unnecessary second-line antibiotic treatment.

Previous studies have demonstrated discordance of vancomycin MICs between different methods (Swenson et al., 2009; Kruzel et al., 2011). Our study suggested 100% essential agreement of both the *E*-test and Vitek II methods with the reference standard, which echoed the findings of Swenson et al. (2009). Vitek II tended to overestimate the measurement in isolates with high MICs (1 and 2 µg/mL), whereas *E*-test tended to underestimate the actual MICs in isolates with a MIC of 0.5 µg/mL. Although previous studies have stated that *E*-tests over estimated high vancomycin MICs and Vitek II might fail to detect vancomycin MIC elevation (Swenson et al., 2009; Bloomgren & Laible, 2013; Tomczak et al., 2013), our results revealed that the *E*-test derived MICs were highly consistent with those provided by broth microdilution. This finding is in line with recent studies (Van Hal et al., 2011; Song et al., 2017). Likewise, Hsu et al. (2008) suggested that MICs derived by *E*-tests were more reflective of the prognosis. However, in this study, we observed noticeable overestimated MICs in Vitek II measurement at a MIC of ≥1 µg/mL. On the other hand, Chen et al. (2014) revealed that vancomycin MICs measured by agar dilution and *E*-test, but not Vitek II, were predictive in terms of clinical outcomes among patients with MRSA bacteremia However, our outcomes are not as optimistic when vancomycin MIC >1 mcg/mL, given that MIC discrepancy between 1–2 is still of interest as variance in MIC determination will affect AUC/MIC exposure calculations (Chen et al., 2014). We speculate that a weaker correlation between “actual” MICs and those detected by Vitek II, compared to *E*-tests, could be the rationale, particularly in hospital-acquired isolates (R coefficient: 0.785 vs. 0.868).

A manuscript closely related to our work was by Phillips et al. which explored the diagnostic accuracy of *E*-test and Vitek II, but used BMD as the gold standard. Similarly, both of our works reached the conclusion that there was a weak level of agreement among these tests, however, Phillips et al. concluded that MIC values tended to be higher when compared against Vitek II and BMD. The authors go on to suggest clinicians in evaluating test results, where, based on the MRSA strains used in their study the optimum *E*-test and Vitek 2 cutoff points for reduced susceptibility was achieved at ≥1 µg/mL. BMD MIC ≥1 µg/mL, the gold standard in their study, has been associated with poor clinical outcomes (Phillips et al., 2016).

**Figure 4:**
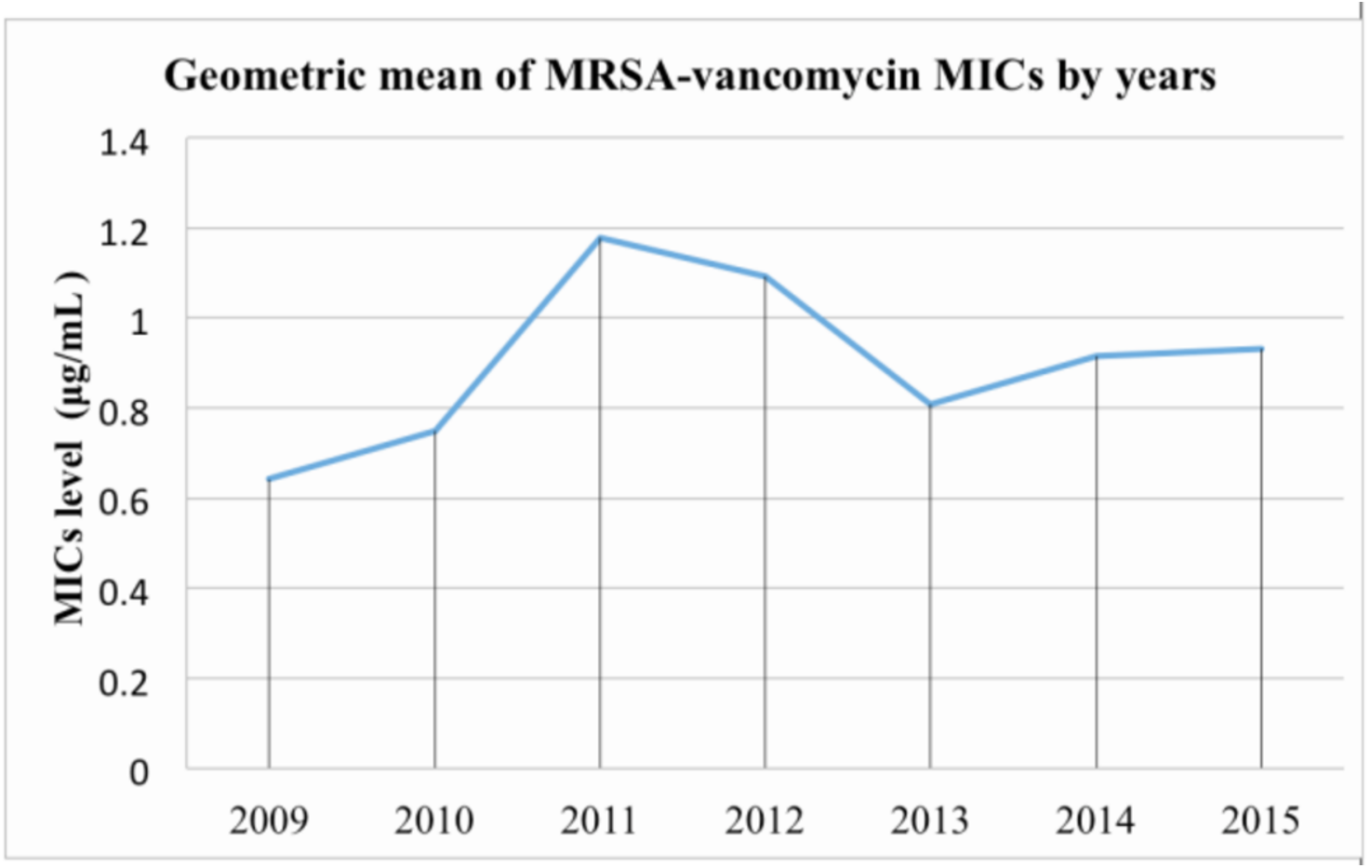
Geometric mean of MRSA-vancomycin MICs by year in a tertiary medical center. The level of MICs level was steadily increasing and there is a vancomycin “Creep” in 2011. The exact etiology of “MIC Creep” is not known; it may due to in-hospital spread of resistant strains or technical artifact caused by applying different measurements. The above MICs were measured by VITEK-II.

Another factor contributing to decreased accuracy of MIC levels across both methods could be due to a relatively recent phenomenon, the “vancomycin creep”. MIC levels have been reported to be steadily increasing worldwide [16, 17], with a significant peak observed in 2011 (Fig. 4). Our finding (Fig. 3) echoes those of recent studies that have addressed this “MIC creep” phenomenon in hospital-acquired MRSA (Haque et al., 2010; Choi et al., 2011), which in turn may lead to decreased accuracy in MIC measurement via both methods (Table 4). However, the exact etiology of the “MIC creep” is still unexplored; it may be either caused by the in-hospital spread of resistant strains or technical artifact brought about by the application of different measurements [15].

Vitek II applies susceptibility cards with different vancomycin concentrations to calculate bacterial growth status in a short period. The overestimation of MICs in Vitek II is notable in this study. One previous study (Chen et al., 2014) speculated that the MIC discrepancy was due to the mathematical adaption of the interpolation method in the Vitek II system, which was not ordinarily recommended by the CLSI. In addition, a study suggested that the high sugar status or biofilm produced by pathogens could disturb the entry of antibiotics and thus affect the procedure of the automated machine. Thus, an overestimation of MIC may be observed (Mottola et al., 2016). Consequently, results obtained at a MIC of 2 µg/mL should be interpreted with caution, in consideration of potential technical errors due to the systemic detection limitation.

Substantial research has indicated that inappropriate antibiotics use could result in the selection of resistant organisms, which might lead to treatment failure. Additionally, frequent alteration of antibiotic formulary could accelerate the development of resistant pathogens. According to the antibiotics stewardships from the Infectious Diseases Society of America (IDSA) for MRSA treatment guideline (Liu et al., 2011), alternative antimicrobial agents of class A-III antibiotics should be adopted when the vancomycin MIC is ≥ 2 µg/mL, because a higher vancomycin dosage might lead to renal toxicity (Brink, 2012). For example, clindamycin, TMP-SMX, linezolid, daptomycin, doxycycline, and tigecycline are all effective alternative treatments (Liu et al., 2011; Campbell et al., 2012). Our data suggests that the use of automated machines may yield inaccurate results when the vancomycin MICs reach 2 µg/mL. Although the rates of resistance to non-glycopeptides for MRSA remain low (Molina & Huang, 2016), the implementation of antimicrobial stewardship programs is crucial for delaying the emergence of resistant strains. Rapid and accurate identification of resistance profiles is a key strategy. Hence, when physicians plan to change treatment for patients with MRSA bacteremia, they should incorporate this information and consider a complementary test to verify the decision of shifting vancomycin to second-line antibiotic treatment.

A discordance in our study is defined as the misclassification of a high MRSA-vancomycin MIC isolate as a low-MIC isolate by using an automated testing method compared with standard broth microdilution. Accordingly, the discordance rate was found to be 0% in Vitek II and 13.3% in *E*-test. This result is not unexpected because Vitek II tends to overestimate at a MIC cutoff of 2 µg/mL, leading to a lower positive predictive value (53.6% vs. 86.7%) and specificity (93.5% vs. 99.0%) than those in *E*-test (Table 5). Therefore, MRSA-vancomycin MICs at a cutoff of 2 µg/mL obtained using Vitek II exhibited a higher sensitivity level (100% vs. 86.7%) and negative predictive value (100% vs. 99.0%) than those obtained using *E*-test in the prediction of categorical agreement with standard broth microdilution.

**Table 5:** Performance measures of high vancomycin MIC value by automated methods in predicting agreement on those obtained by standard microdilution method among 216 patients with MRSA bacteremia.

		**MIC ≥ 2** (*μ* g/mL)	**MIC <2** (*μ* g/mL)	**Sensitivity (%)**	**Specificity (%)**	**PPV (%)**	**NPV (%)**
VITEK-II	MIC ≥ 2 (µg/mL)	15	13	100.00%	93.53%	53.57%	100.00%
	MIC <2 (µg/mL)	0	188				
*E*-test	MIC ≥ 2 (µg/mL)	13	2	86.67%	99.00%	86.67%	99.00%
	MIC <2 (µg/mL)	2	199				

**Notes.**

MIC minimum inhibitory concentration MRSA methicillin-resistant Staphylococcus aureus PPV positive predicative value NPV negative predictive value

Definition of PPV and NPV detected by VITEK-II and *E*-test: PPV = TP / (TP + FP) = MIC ≥ 2 / (MIC ≥ 2 + MIC <2) NPV = TN / (FN + TN) = MIC <2/ (MIC ≥ 2 + MIC <2).

The limitations of this study mainly comprised of the following: first, as a single hospital-based study, the generalizability of the results would require further validation. Second, the MRSA strains in this study was stored for more than 12 months after isolation, which a report suggested that frozen storage significantly affects the MICs in MRSA isolates (Edwards et al., 2012). Third, we did not collect information on clinical prognosis. Fourth, we did not do controls for BMD. The literature contains conflicting data regarding whether MRSA-vancomycin MICs could actually reflect the prognosis. Some studies have suggested that vancomycin MICs enabled the determination of adverse clinical outcomes (Honda et al., 2011; Han et al., 2012; Rojas et al., 2012; Hope et al., 2013), whereas others have not (Hos et al., 2017; Song et al., 2017; Adani et al., 2018; Bouiller et al., 2018). Because the MIC-guided treatment decision is controversial in low MRSA-vancomycin MICs (≤2 µg/mL), the IDSA also recommends that the actual clinical and microbiological response should be considered as a guide to treatment decisions if the MRSA-vancomycin MIC is low (Liu et al., 2011). A prospective study is warranted to further clarify the clinical prognosis of different treatment schemes based on various MIC measurement methods.

Nonetheless, treatment of MRSA infections should still be primarily based on clinical response to vancomycin rather than MIC levels alone, as the efficiency of antibiotics is not only based on susceptibility or resistance via MIC levels but should also take into consideration renal or hepatic functions, drug penetration, efficiency and metabolism. Thus, a better practice would be reviewing patients’ profiles in conjunction to referencing MIC levels. In patients with impaired renal function, for example, antibiotics with renal toxicity such as vancomycin should not be considered as the drug of choice. Different antibiotics should reflect pharmacodynamics and pharmacokinetics and the type of infection, i.e., community- acquired pneumonia, hospital- acquired pneumonia, or various skin and soft tissue infections, and their application should be used in accordance with local guidelines.

## Conclusion

The results of this study provide an insight to the discrepancy between MICs measured via Vitek II and those measured with other methods at a cutoff of 2 µg/mL may imply clinical significance. MRSA-vancomycin MICs measured with automated methods must be interpreted warily. When physicians adopt MRSA-vancomycin MICs to guide their decision regarding the treatment scheme, they should consider another complementary test for the confirmation, particularly for those results that are very close to the cutoff value. This may thus reduce the inappropriate use of antibiotics and the emergence of resistant strains.

##  Supplemental Information

10.7717/peerj.8963/supp-1 Supplemental Information 1 The Vancomycin minimum inhibitory concentration for Methicillin-Resistant Staphylococcus aureus between Vitek II, *E*-test, and Broth Microdilution Each data point indicates the Vancomycin minimum inhibitory concentration for Methicillin-Resistant Staphylococcus aureus. (A) column 1: Infection source 1 = hospital-acquired infection; 0 = Community-acquired infection; (B) column 2: VitekII: MIC level; (C) column 3: *E*-test: MIC level; (D) column 4: Microdilution: MIC level.

## Additional Information and Declarations

### Data Availability

The following information was supplied regarding data availability: The raw measurements are available as a Supplementary File.
